# Supplementary material for: A Compact Quadrupole-Orbitrap Mass Spectrometer with FAIMS Interface Improves Proteome Coverage in Short LC Gradients
Source: Mol Cell Proteomics. 2020 Feb 12;19(4):716–29. doi: 10.1074/mcp.TIR119.001906 (PMC7124470; doi:10.1074/mcp.TIR119.001906)
Supplement: Supplementary Information [file 157671_1_supp_469134_q54ww1.pdf]

Supplementary Figure 1

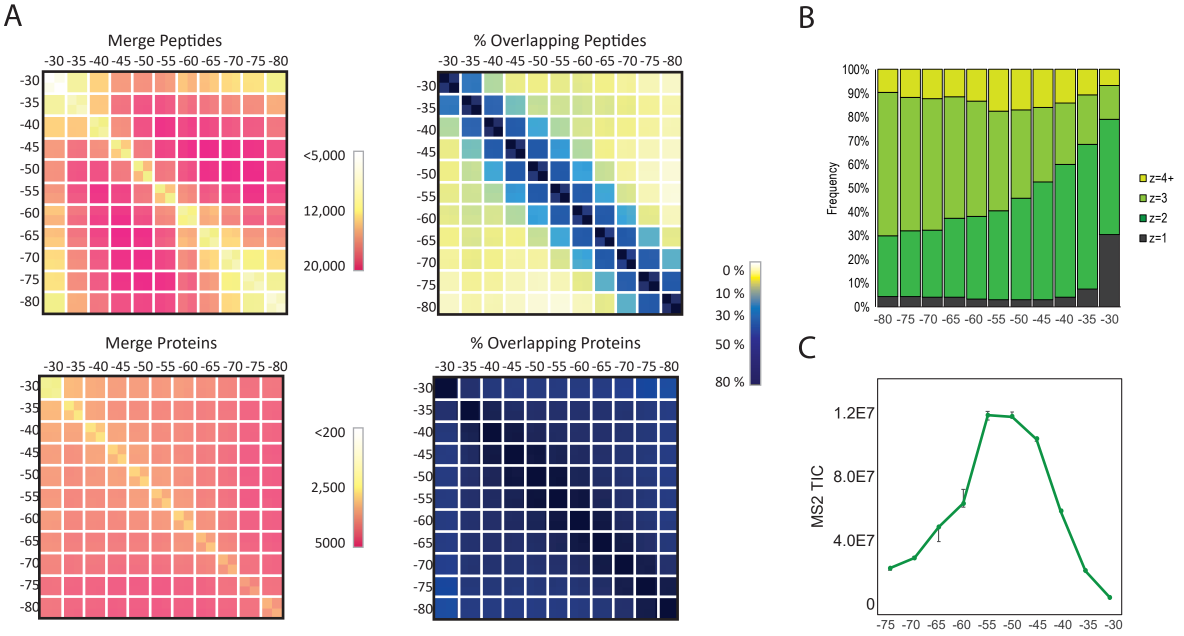

Supplementary Figure 1. (a) Heatmap showing the number of peptides or proteins gained by merging independent DDA-FAIMS runs with different compensation voltages. On the right, heatmap showing the percentage of common peptides or proteins identified in independent DDA-FAIMS runs with different compensation voltages. **(b)** Precursor charge distribution using different compensation voltages. **(c)** MS2-TIC average values at different compensation voltages from 500 ng peptide in a 21 minutes gradient using DDA settings.

Supplementary Figure 2

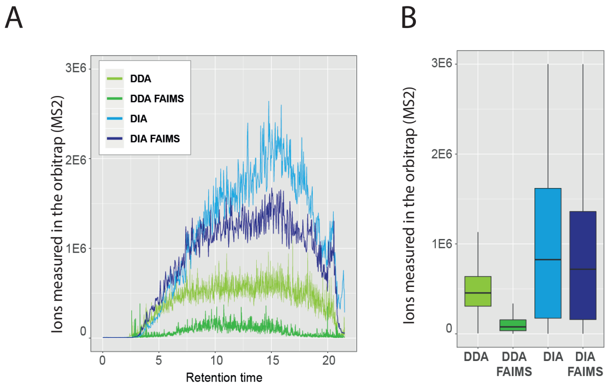

Supplementary Figure 2. (a) Comparison of MS2 ion intensity levels across time in DDA, DDA-FAIMS, DIA and DIA-FAIMS runs using 500 ng peptide. **(b)** Boxplot showing the MS2 ion intensity distribution in DDA, DDA-FAIMS, DIA and DIA-FAIMS runs using 500 ng peptide.

Supplementary Figure 3

A

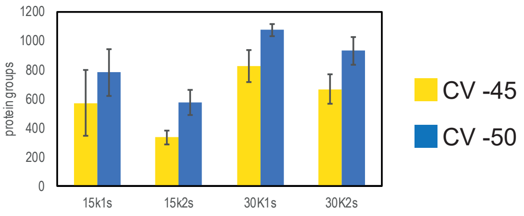

B

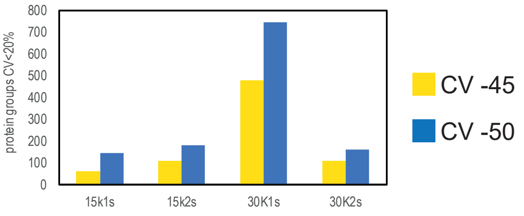

Supplementary figure 3. (a) Protein groups identified in total using different acquisition methods, cycle times and CV in DIA-FAIMS mode using 5 ng of peptide in a 5 minute gradient (200 samples per day). **(b)** Proteins with a coefficient of variation below 20% for the same parameters as in 3a.

Supplementary Figure 4

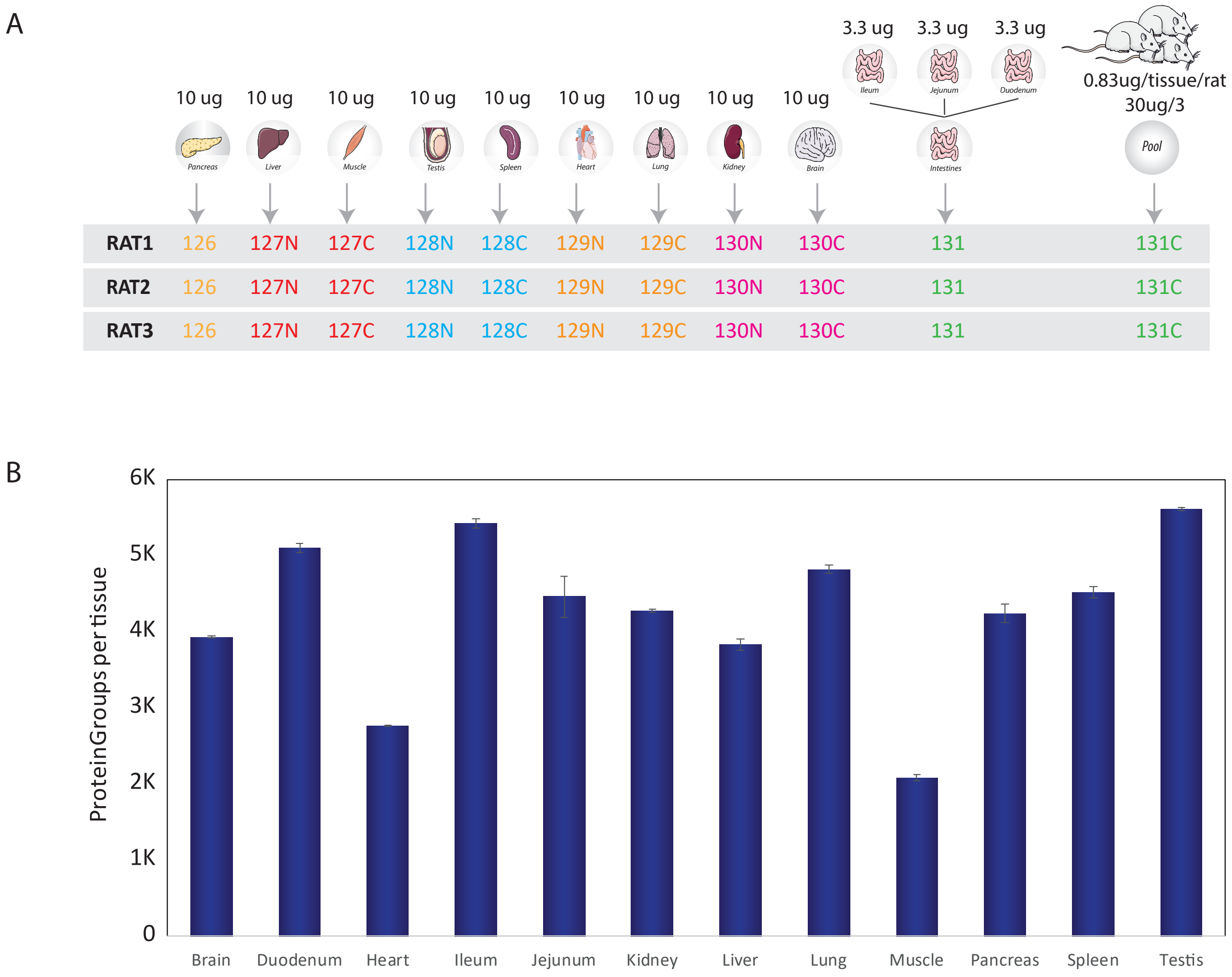

Supplementary figure 4. (a) TMT labeling scheme for full proteome analysis. **(b)** Bar chart showing the number of protein groups per tissue quantified in each independent tissue in DIA-FAIMS analysis. Plotted value is the average of three replicates, and the error bars represent the standard deviation among them.

Supplementary Figure 5

A

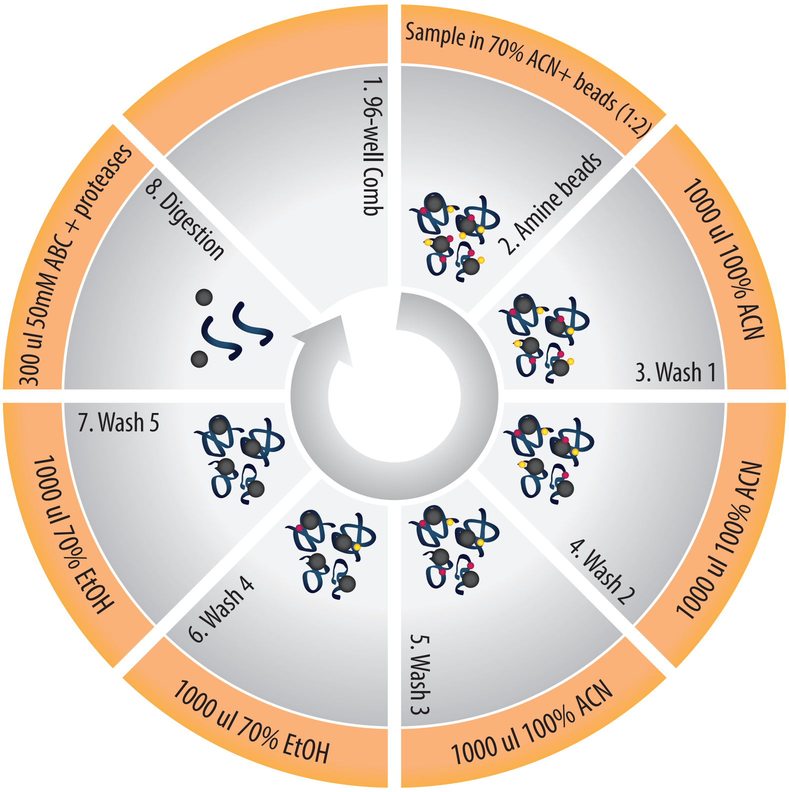

B

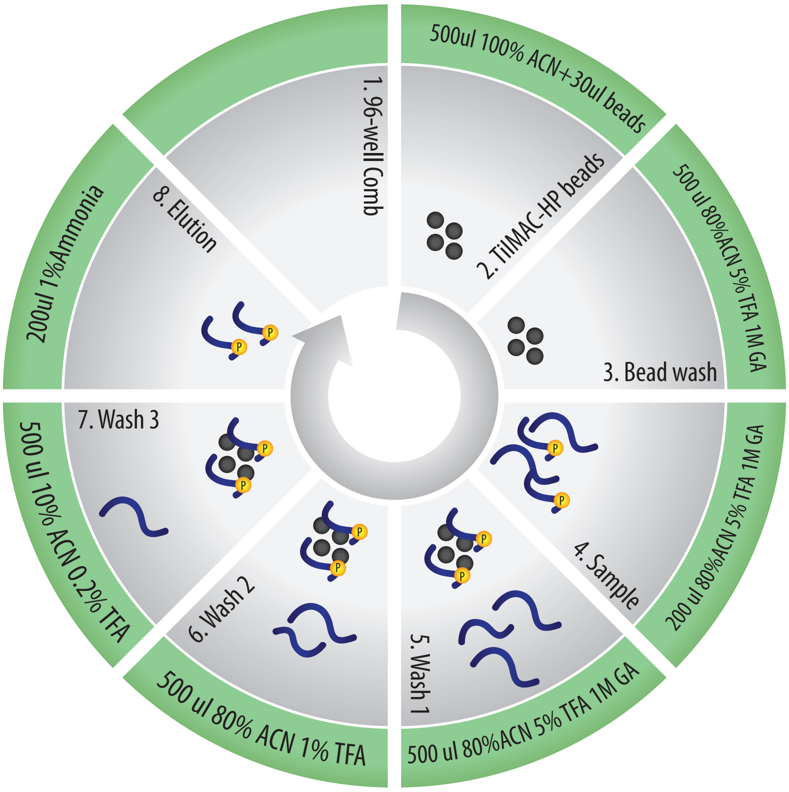

Supplementary figure 5. (a) Detailed PAC digestion workflow on the KingFisher Flex robot. **(b)** Detailed phospho-enrichment workflow on the KingFisher Flex robot.
